# Elderly Caribbean Hispanic men have lower tibial stiffness and failure load compared to non-Hispanic White and Black men

**DOI:** 10.1093/jbmrpl/ziaf110

**Published:** 2025-06-24

**Authors:** Sanchita Agarwal, Ragyie Rawal, Carmen Germosen, Isabella Rosillo, Lynette X Lopez, Ivelisse Colon, Mariana Bucovsky, Mildense Del Orbe, X Edward Guo, Elizabeth Shane, Marcella Walker

**Affiliations:** Division of Endocrinology, Department of Medicine, Columbia University Irving Medical Center, New York, NY 10032, United States; Division of Endocrinology, Department of Medicine, Columbia University Irving Medical Center, New York, NY 10032, United States; Division of Endocrinology, Department of Medicine, Columbia University Irving Medical Center, New York, NY 10032, United States; Division of Endocrinology, Department of Medicine, Columbia University Irving Medical Center, New York, NY 10032, United States; Bone Bioengineering Laboratory, Department of Biomedical Engineering, Columbia University, New York, NY 10027, United States; Division of Endocrinology, Department of Medicine, Columbia University Irving Medical Center, New York, NY 10032, United States; Division of Endocrinology, Department of Medicine, Columbia University Irving Medical Center, New York, NY 10032, United States; Division of Endocrinology, Department of Medicine, Columbia University Irving Medical Center, New York, NY 10032, United States; Bone Bioengineering Laboratory, Department of Biomedical Engineering, Columbia University, New York, NY 10027, United States; Division of Endocrinology, Department of Medicine, Columbia University Irving Medical Center, New York, NY 10032, United States; Division of Endocrinology, Department of Medicine, Columbia University Irving Medical Center, New York, NY 10032, United States

**Keywords:** race, microstructure, ethnicity, Hispanic, stiffness

## Abstract

No data exist comparing racial differences in bone microstructure or mechanical competence using HR-pQCT and micro-finite element analysis (μFEA) in elderly Hispanic, non-Hispanic Black (NHB), and non-Hispanic White (NHW) men. These modalities were utilized to investigate skeletal health in 255 elderly men (age ≥ 65) from a population-based study in New York City: 40.0% Caribbean Hispanic (CH), 35.3% NHW, and 24.7% NHB. Covariate-adjusted (age, BMI, calcium consumption, smoking, diabetes, liver disease, and HIV) results are shown. We also explored the effect of socioeconomic (SE) factors. At the distal tibia, CH men had lower trabecular indices with 9% lower stiffness and failure load (both *p* < .05) compared to NHW. CH men had smaller cortical area (Ct.Ar) and lower thickness (Ct.Th) compared to NHB, with 11% (*p* < .05) lower stiffness and failure load. After adjusting for SE, differences in stiffness and failure load between CH and NHW were no longer significant. Comparing NHB to NHW men at the tibia, NHB had lower trabecular indices but greater Ct.Ar, Ct. volumetric bone density (Ct.vBMD) and Ct.Th, with no differences in stiffness and failure load. At the diaphyseal tibia, Ct.Ar and Ct.Th were lower in CH compared to both NHW and NHB men, with 11% and 17% lower stiffness and failure load compared to NHW and NHB (all *p* < .05). Radial μFEA indices were not different. In conclusion, CH elderly men have lower mechanical competence at the tibia compared to NHW and NHB men, which could result in a greater risk of incident fracture in CH men. Some differences between CH and NHW may be related to modifiable SE factors. Studies assessing HR-pQCT’s ability to predict incident fracture and how SE factors affect fracture risk are needed in men of races and ethnicities historically underrepresented in skeletal research.

## Introduction

Bone mineral density and fracture incidence vary by race and ethnicity within the United States. Areal BMD (aBMD) measured by DXA tends to be higher, while fracture risk is lower, in NHB older adults as compared to NHW.^1^ Asians have lower aBMD, but also lower risk for some types of fractures. Data in Hispanics are more variable. Hispanic compared to NHW individuals have been shown to have higher, lower, or similar aBMD and fracture risk, depending on the study.^1–8^ These differences may relate to country of origin, skeletal site, or both.^9^ Racially diverse US cohorts with skeletal outcomes are extremely limited and most of the available data have been acquired in women.^9^^,^^10^ Data in men are incomplete. This information is needed in order to better understand fracture risk and risk factors in our increasingly aging and diverse population.

High-resolution peripheral quantitative computed tomography has uncovered racial and ethnic differences in bone microstructure and mechanical competence not evident with DXA.^11^^,^^12^ These differences could contribute to racial variation in fracture risk.^11^^,^^12^ By combining HR-pQCT data from 8 cohort studies that included 7254 predominantly NHW participants, a recent analysis found that failure load was more strongly associated with incident fractures than FN areal bone density.^13^ In a population-based cohort of older adults, we recently reported that although CH women had better cortical parameters than NHW women, failure load was similar.^14^ In contrast, CH women had worse trabecular and cortical parameters than NHB women, and failure load was lower. No data regarding volumetric bone density or bone microstructure from HR-pQCT are available in Hispanic or NHB elderly men.

In this multi-ethnic, population-based study, we aimed to evaluate and compare the skeletal health of elderly (age ≥ 65) CH men to NHW and NHB men using HR-pQCT, micro-finite element analysis (μFEA), and individual trabecula segmentation (ITS).^15^ We hypothesized that men of CH descent would have lower failure load than NHB men, similar to our findings in CH women.^14^

## Materials and methods

### Study design

This cross-sectional ancillary study collected skeletal health data from elderly CH, NHW, and NHB men from a prospective population-based aging study. The Columbia University Irving Medical Center Institutional Review Board approved this study. All study participants gave written informed consent.

### Study population

The Washington Heights Hamilton Heights Inwood Community Aging Project is a population-based longitudinal study of aging in older, city-dwelling ambulatory residents (age ≥ 65) who reside in Northern Manhattan. Study design and enrollment procedures have been previously reported.^14^^,^^16^^,^^17^ Briefly, residents in 3 zip codes in New York City were randomly invited to participate if they were age 65 or older, did not have dementia, and enrolled in Medicare. Participants able to consent were invited to this ancillary bone health study for assessment with DXA and HR-pQCT. Medical history was acquired by questionnaire. Though all races and ethnicities were invited to participate, most individuals were of CH, NHB, and NHW due to the location of the study. In this cross-sectional analysis, we included men enrolled to the ancillary study who self-identified as NHB (*n* = 63), NHW (*n* = 90), and CH (*n* = 102). Imaging was obtained between January 2019 and March 2024. Due to fewer numbers, other races/ethnicities and Hispanic subgroups were not included in the analysis.

### DXA

A Hologic densitometer was used to measure areal BMD at the lumbar spine (LS; L1-L4), FN, TH, and distal 1/3 radius. Participants were scanned at all skeletal sites unless artifacts were present. The manufacturer’s White reference database was used to calculate *T*-scores. Vertebra with hardware or other artifacts were excluded from BMD spine analysis. Our DXA precision is 1.28%, 1.36%, and 0.70% at the spine, hip, and 1/3-radius.^18^

Medimaps software (version 3.1.2)^19^ was used to calculate the trabecular bone score (TBS) from measurable spine DXA images. We obtained lateral vertebral fracture assessment (VFA) from T4 to L5. The Genant visual semi-quantitative method categorized participants as having mild, moderate, or severe vertebral compression fractures, defined as 20%-25%, 26%-40% or >40% height loss, respectively.^20^

### HR-pQCT

An XtremeCT II scanner (Scanco Medical) with 68 kVp voltage, 1460 μA current, 43 s integration time, and a 60.7 μm isotropic voxel size was used. One operator obtained and analyzed all scans. The non-dominant distal radius and tibia were routinely scanned. The dominant side was used if a previous fracture or hardware was present. To delineate the region of interest, a line was placed on a 2-D scout view at the endplate (proximal for the radius, distal for the tibia). We used a relative offset from the reference line to obtain radius and tibia scans at 4% and 7.3% of the limb length, respectively. The axial scan region was 10.2 mm long. A tibial diaphyseal cortical site (30%) was also scanned. Any scans with motion scores >3 (using a scale 1-5) were discarded.^22^ The manufacturer’s method was used to filter and binarize the HR-pQCT images. An automated segmentation algorithm separated the cortical and trabecular regions.^21^ Standard HR-pQCT measures were obtained: total, trabecular (Tb), and cortical (Ct) area and volumetric BMD (vBMD); microstructure—trabecular number (Tb.N), thickness (Tb.Th), separation (Tb.Sp), standard deviation of the trabecular separation (Tb.1/N.SD), cortical thickness (Ct.Th), cortical porosity (Ct.Po), and pore size (Ct.Po.Dm). Reproducibility in our lab is 0%-5% for all parameters except Ct.Po, which is 9.9%-15.6% at distal tibia and radius.^22^

### Finite element analysis

A voxel-conversion-based μFEA approach was used to assess bone strength from HR-pQCT images. We computer-simulated an axial compression on each radius and tibia model up to 1% strain using a homogeneous Young’s modulus of 10 GPa and Poisson’s ratio of 0.3 to estimate whole bone stiffness. The Pistoia criterion^23^ was used to estimate failure load. The manufacturer’s μFEA solver (Scanco Medical FE-software v1.13, Scanco Medical) was employed.

### Individual trabecula analysis

Individual trabecula segmentation is a validated method, which decomposes the cancellous bone network into individual plates and rods from second generation HR-pQCT images.^24–26^ Plate and rod number density (pTb.N and rTb.N, 1/mm; cubic root of total number of plates or rods/bulk volume), plate-plate junction density (P-P Junc.D, 1/mm^3^; total number of junctions/bulk volume), and plate-rod junction density are reported.

### Questionnaire and clinical evaluation

We collected data from participants about past medical history, medications, lifestyle, falls, calcium, and vitamin D consumption, and physical activity via questionnaires.^27^^,^^28^ A balance beam scale and a wall-mounted, calibrated Harpenden stadiometer measured weight and height, respectively. Osteoporotic fractures were defined as non-traumatic or low trauma (fall from standing height or less) adult fractures. We excluded fractures of the fingers, toes, skull, and face. Osteoporotic non-vertebral fractures included osteoporotic fractures except vertebral fractures.

### Statistics

We report descriptive statistics as either means and standard deviations or absolute (*n*) and relative (%) frequency. Statistical significance was defined as a 2-tailed *p*-value <.05. Between-group differences were evaluated using one-way analysis of variance with post-hoc Tukey’s test or Pearson’s chi-square test with post-hoc 2×2 contingency table comparisons. Analysis of covariance was used for adjusted analysis, controlling for age, BMI, calcium consumption, tobacco use, diabetes, liver disease, and human immunodeficiency virus or HIV (model 1). Additional models adjusting for education and household income in addition to the prior covariates were also explored (model 2).^14^ Covariates were chosen due to known associations with bone health and/or between-group differences. We used Bonferroni correction to account for multiple comparisons between highly correlated trabecular HRpQCT parameters (density, number, thickness, and separation).^29^^,^^30^ We also explored adjusting for weight and height despite acquiring scans using a relative offset in place of BMI. All analyses were performed using Python (version 3.7.4) and R (version 3.6.3).

## Results

### Demographic and skeletal risk factors

Of 255 men included in this analysis, 40.0% were CH, 35.3% were NHW and 24.7% NHB. Mean age of the cohort was 76.0 ± 5.8 yr. Compared to NHW men, CH men were shorter, had lower calcium intake, were more likely to have diabetes and be immigrants, and had a lower education and income level (Table 1). Compared to NHB, CH men were shorter, weighed less, were less educated, tended to be immigrants, had lower income and were less likely to have HIV or liver disease. Compared to NHW, NHB men were slightly younger, taller, and had lower education and income level, as well as lower calcium intake (Table 1). They were also more likely to be smokers and have HIV, but less likely to have a family history of hip fracture. As shown in Table 1, there were no differences between groups in BMI, vitamin D intake, osteoporotic fractures, falls, exercise, alcohol use, osteoporosis treatment, or glucocorticoid use.

**Table 1:** Racial/ethnic differences in demographic and clinical characteristics.

**Characteristic**	**NHB (*n* = 63)**	**NHW (*n* = 90)**	**CH (*n* = 102)**	***p***
**Age (yr)**	74.4 ± 5.6	76.8 ± 4.6	76.2 ± 6.6	**.04** ^**a**^
**Height (inches)**	69.5 ± 2.5	67.8 ± 2.9	65.9 ± 2.3	**<.001** ^**a**^ ^**-**^ ^**c**^
**Weight (pounds)**	191.2 ± 39.5	181.7 ± 33.0	174.1 ± 29.1	**.01** ^**c**^
**BMI (kg/m** ^**2**^ **)**	27.8 ± 5.5	27.8 ± 4.7	28.1 ± 4.1	.85
**Place of birth (%)**				**<.001** ^**b**^ ^**,**^ ^**c**^
**United States**	98.4	88.9	2.9	
**Outside United States**	1.6	11.1	97.1	
**Highest education level (%)**				**<.001** ^**a**^ ^**-**^ ^**c**^
**Grammar school**	7.9	1.1	51.0	
**High school**	55.6	11.1	34.3	
**College or advanced professional degree**	36.5	87.8	14.7	
**Household income (%)**				**<.001** ^**a**^ ^**-**^ ^**c**^
**<$50 k**	77.4	28.2	96.0	
**$50 k to <$200 k**	21.0	57.6	4.0	
**>$200 k**	1.6	14.1	0.0	
**Total daily calcium intake (mg/d)**	1103 ± 584	1311 ± 566	939 ± 467	**<.001** ^**a**^ ^**,**^ ^**b**^
**Total daily vitamin D intake (IU/d)**	1926 ± 6263	1714 ± 1620	1234 ± 1664	.40
**Osteoporotic fractures (%)**	13	10	8	.59
**Nonvertebral osteoporotic fractures (%)**	13	10	8	.59
**Percent with fall in past 12 mo (%)**	22	24	19	.61
**Falls in past 12 mo (*n*)**	1.4 ± 0.6	2.1 ± 2.4	2.2 ± 2.4	.50
**Family history of hip fracture (%)**	3	16	11	.06
**Exercise (PASE score)**	92.7 ± 50.1	83.1 ± 48.3	84.1 ± 53.5	.47
**Current smokers (%)**	16	2	6	**.004** ^**a**^
**Alcohol intake (drinks/wk)**	2.9 ± 8.5	4.2 ± 6.9	2.2 ± 4.3	.10
**Liver disease (%)**	14	6	3	**.02** ^**c**^
**HIV (%)**	8	0	0	**<.001** ^**a**^ ^**,**^ ^**c**^
**Diabetes (%)**	24	16	39	**<.001** ^**b**^
**Current osteoporosis treatment (%)**	0	1	3	.31
**Past osteoporosis treatment (%)**	2	4	2	.47
**Current oral glucocorticoid use (%)**	0	2	0	.29

Data are shown in mean ± SD or %. Values of *p* < .05 were considered significant and are shown in bold.

a *p* < .05 NHB vs NHW.

b *p* < .05 CH vs NHW.

c *p* < .05 CH vs NHB.

Abbreviations: CH, Caribbean Hispanic; NHB, non-Hispanic Black; NHW, non-Hispanic White.

### Areal BMD by DXA

**Figure 1:**
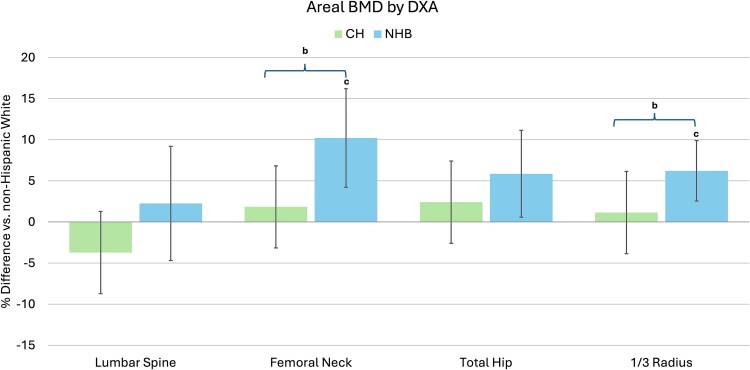
Adjusted racial/ethnic differences in areal BMD by DXA in CH (green) and NHB (blue) compared to NHW men; ^a^*p* < .05 CH compared to NHW; ^b^*p* < .05 CH compared to NHB; ^c^*p* < .05 NHB compared to NHW.

Comparing CH to NHW men, aBMD did not differ before or after adjustment of covariates (Table 2 and Figure 1). Compared to NHB men, CH men had 4.8% lower aBMD at the 1/3 radius (*p* < .001), but no differences at other sites. After adjusting for age, BMI, calcium intake, smoking, diabetes, liver disease, and HIV (model 1), this difference persisted, and FN aBMD was also lower in CH men. Compared to NHW men, NHB men had 6.4% and 9.8% higher aBMD at the 1/3 radius (*p* < .001) and FN, respectively, (*p* = .002) prior to adjustment for covariates. Differences at the TH were of borderline significance (*p* = .04). After adjusting for age, BMI, calcium intake, smoking, diabetes, liver disease, and HIV, NHB compared to NHW had higher adjusted aBMD at the FN and 1/3-radius [model 1 (*p* < .001)]. Differences at the TH were of borderline significance (*p* = .06). There were no differences at the spine before or after adjustment for covariates (Figure 1). Adjusting for height and weight instead of BMI (Table S1), aBMD results were similar.

**Table 2:** Racial/ethnic differences in aBMD, VFA, and TBS by DXA.

**Parameter**	**NHB (*n* = 63)**	**NHW (*n* = 90)**	**CH (*n* = 102)**	***p***	**Adjusted *p*** ^**a**^	**Adjusted *p*** ^**b**^
**LS *T*-score**	0.4 ± 2.0	0.4 ± 1.8	0.1 ± 1.7	.59	.16	.31
**FN *T*-score**	−0.7 ± 1.1	−1.3 ± 0.9	−1.1 ± 1.1	**.002** ^**c**^	**<.001** ^**c**^ ^**,**^ ^**d**^	**<.001** ^**c**^ ^**,**^ ^**d**^
**TH *T*-score**	−0.5 ± 1.0	−0.8 ± 0.9	−0.5 ± 1.0	**.04** ^**f**^	.06	.05
**1/3-Radius *T*-score**	−0.3 ± 1.6	−1.3 ± 1.4	−1.0 ± 1.4	**<.001** ^**c**^ ^**,**^ ^**d**^	**<.001** ^**c**^ ^**,**^ ^**d**^	**<.001** ^**c**^ ^**,**^ ^**d**^
**Percent with ≥1 spine fracture(s) detected by VFA (%)**	10	11	15	.55	.25	.31
**Number of spine fractures in those with ≥1 spine fracture(s) (*n*)**	1.5 ± 0.8	1.1 ± 0.3	1.1 ± 0.4	.24	.69	.15
**TBS**	1.3 ± 0.1	1.3 ± 0.1	1.3 ± 0.1	.21	.37	.86

Data are shown as mean ± SD or %. Values of *p* < .05 were considered significant and are shown in bold.

Abbreviations: CH, Caribbean Hispanic; NHB, non-Hispanic Black; NHW, non-Hispanic White.

a Model adjusted for age, BMI, calcium intake, smoking, diabetes, liver disease, and HIV.

b Model adjusted for age, BMI, calcium intake, smoking, diabetes, liver disease, HIV, education, and household income.

c *p* < .05 NHB vs NHW.

d *p* < .05 CH vs NHB.

e *p* < .05 CH vs NHW.

f No pairwise Tukey comparisons had *p* < .05.

### VFA and TBS by DXA

Prevalence of vertebral fracture by VFA was similar across the racial groups both before and after adjustment for covariates. Trabecular bone score did not differ across the groups before or after adjustment of covariates as well (Table 2).

### HR-pQCT

#### Distal radius

**Figure 2:**
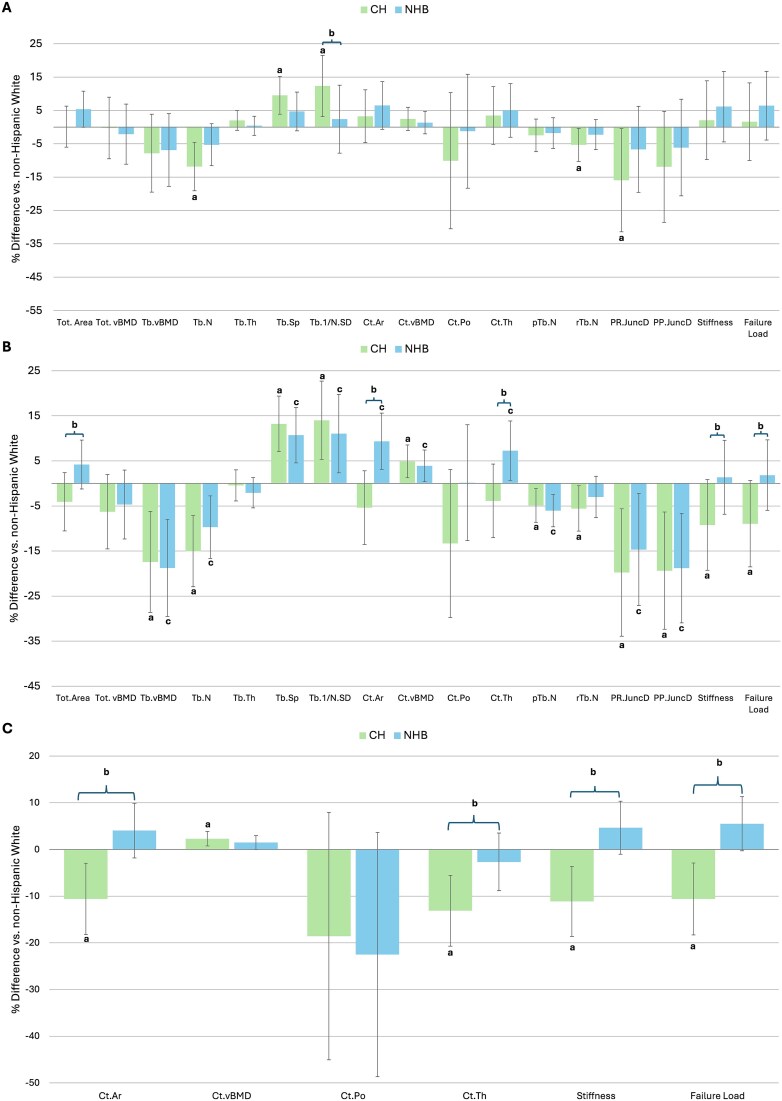
Adjusted racial/ethnic differences in HR-pQCT and FEA indices at the (A) radius, (B) distal tibia, and (C) diaphyseal tibia in CH (green) and NHB (blue) compared to NHW men; ^a^*p* < .05 CH compared to NHW; ^b^*p* < .05 CH compared to NHB; ^c^*p* < .05 NHB compared to NHW.

Table 3 and Figure 2A indicate differences in radial skeletal microstructure by HR-pQCT. Compared to NHW, CH men had 10.0% fewer (*p* < .001), 2.5% thicker (*p* = .04), 10.2% more widely spaced (*p* < .001), and 14.2% more heterogeneously distributed trabeculae (*p* = .01) before adjustment for covariates. CH men also had 3.2% greater unadjusted cortical density, 4.4% lower trabecular rod number, and 11.7% lower plate-rod junction density compared to NHW (all *p* = .03). After adjusting for age, BMI, smoking, diabetes, liver disease, and HIV (model 1), differences in Tb.N, Tb.Sp, Tb. heterogeneity, Tb. rod number, and plate-rod junction density persisted (Figure 2A). Stiffness and failure load were not different before or after adjustment comparing CH and NHW men.

**Table 3:** Racial/ethnic differences in vBMD, microstructure, and estimated mechanical competence by HR-pQCT at the distal radius scanned at 4% relative offset.

**Parameter**	**NHB (*n* = 63)**	**NHW (*n* = 90)**	**CH (*n* = 102)**	***p***	**Adjusted *p*** ^**a**^	**Adjusted *p*** ^**b**^
**Tt.Ar (mm** ^**2**^ **)**	387 ± 59	366 ± 63	360 ± 49	**.01** ^**c**^	.10	.13
**Tot.vBMD (mg HA/cm** ^**3**^ **)**	277 ± 61	282 ± 59	289 ± 64	.42	.84	.34
**Tb.vBMD (mg HA/cm** ^**3**^ **)**	153 ± 35	167 ± 37	159 ± 37	.07	.15	.70
**Tb.N (1/mm)**	1.38 ± 0.20	1.48 ± 0.20	1.34 ± 0.21	**<.001** ^**d**^ ^**,**^ ^**e**^	**<.001** ^**d**^	.29
**Tb.Th (mm)**	0.24 ± 0.02	0.24 ± 0.02	0.24 ± 0.02	**.04** ^**d**^	.16	.22
**Tb.Sp (mm)**	0.70 ± 0.12	0.65 ± 0.11	0.72 ± 0.14	**<.001** ^**d**^ ^**,**^ ^**e**^	**<.001** ^**d**^	.31
**Tb.1/N.SD (mm)**	0.27 ± 0.07	0.25 ± 0.07	0.29 ± 0.11	**.01** ^**d**^	**.002** ^**c**^ ^**,**^ ^**d**^	.12
**Ct.Ar (mm** ^**2**^ **)**	72 ± 16	65 ± 14	69 ± 15	**.03** ^**e**^	.17	.10
**Ct.vBMD (mg HA/cm** ^**3**^ **)**	826 ± 75	811 ± 69	837 ± 73	**.04** ^**d**^	.16	.40
**Ct.Po (%)**	1.46 ± 0.89	1.50 ± 0.75	1.37 ± 0.79	.52	.26	.60
**Ct.Th (mm)**	1.04 ± 0.24	0.97 ± 0.21	1.04 ± 0.25	.07	.35	.12
**Tb. Plate number**	1.52 ± 0.17	1.55 ± 0.17	1.53 ± 0.16	.48	.40	.70
**Tb. Rod number**	1.59 ± 0.18	1.62 ± 0.18	1.55 ± 0.17	**.03** ^**d**^	**.02** ^**d**^	.57
**Plate-rod junction density**	4.16 ± 1.23	4.44 ± 1.27	3.97 ± 1.07	**.03** ^**d**^	**.01** ^**d**^	.84
**Plate-plate junction density**	2.75 ± 0.86	2.94 ± 0.93	2.73 ± 0.79	.20	.14	.93
**Stiffness**	77 412 ± 20 674	73 001 ± 20 674	75 817 ± 21 288	.42	.56	.32
**Failure load (N)**	4190 ± 1080	3942 ± 1098	4072 ± 1138	.40	.52	.32

a Model adjusted for age, BMI, calcium intake, smoking, diabetes, liver disease, and HIV.

b Model adjusted for age, BMI, calcium intake, smoking, diabetes, liver disease, HIV, education, and household income.

c *p* < .05 CH vs NHB.

d *p* < 0.05 CH vs NHW.

e *p* < 0.05 NHB vs NHW.

Data are shown as mean ± SD. Values of *p* < .05 were considered significant and are shown in bold.

Abbreviations: HA, hydroxyapatite; CH, Caribbean Hispanic; NHB, non-Hispanic black; NHW, non-Hispanic white.

Compared to NHB, CH men had 7.4% lower unadjusted total area (*p* = .01). After adjustment for age, BMI, calcium intake, smoking, diabetes, liver disease, and HIV (model 1), CH men had 10.2% more heterogeneously distributed trabeculae (*p* = .002) compared to NHB men. Stiffness and failure load were not different comparing CH and NHB men before or after adjustment.

Compared to NHW men, NHB men had 6.8% lower trabecular number and 8.1% higher spacing (*p* < .001), with 9.1% larger cortical area (*p* = .03) before adjustment. After adjustment for covariates, there were no differences between NHW and NHB men (model 1; Figure 2A).

We also explored current use of osteoporosis therapeutics as a covariate in adjusted models. Including osteoporosis medication use in adjusted models did not change the results. Adjusting for height and weight rather than BMI did not change the results (Table S1).

After correcting for multiple comparisons for correlated trabecular parameters (density, number, thickness, and separation), all differences remained significant (*p* < .0125) except for trabecular thickness in the unadjusted model (*p* = .04).

#### Distal tibia

Table 4 and Figure 2B show differences in skeletal microstructure at the distal tibia by HR-pQCT. Compared to NHW, CH men had 15.8% lower unadjusted trabecular density, 15.0% lower trabecular number, and 14.6%-16.7% higher spacing and heterogeneity of the trabecular network (all *p* < .001). CH men also had 18.4% lower cortical porosity (*p* = .01) and 5.7% higher density (*p* < .001). By ITS, CH men had 4.5%-5.3% fewer trabecular plates (*p* < .001) and rods (*p* = .01), with 18.2%-19.6% lower plate-plate and plate-rod junction densities (*p* < .001). Ultimately, this was associated with 7.2% lower failure load (*p* = .03) in CH compared to NHW men before covariate adjustment. After adjustment for age, BMI, calcium intake, diabetes, liver disease, and HIV (model 1), all differences persisted (*p* < .001) except for cortical porosity. Additionally, adjusted cortical density was 4.9% higher (*p* = .002) and trabecular rod number was 5.6% lower (*p* = .01) in model 1 (Figure 2B). Ultimately, CH men had 8.9%-9.2% lower failure load and stiffness (*p* = .01) in model 1 (Figure 2B).

**Table 4:** Racial/ethnic differences in vBMD, microstructure, and estimated mechanical competence by HR-pQCT at the distal tibia scanned at 7.3% relative offset.

**Parameter**	**NHB (*n* = 63)**	**NHW (*n* = 90)**	**CH (*n* = 102)**	***p***	**Adjusted *p*** ^**a**^	**Adjusted *p*** ^**b**^
**Tt.Ar (mm** ^**2**^ **)**	892 ± 135	862 ± 136	824 ± 105	**.003** ^**c**^	**.01** ^**c**^	**.02** ^**c**^
**Tot.vBMD (mg HA/cm** ^**3**^ **)**	275 ± 56	289 ± 48	277 ± 49	.14	.10	.97
**Tb.vBMD (mg HA/cm** ^**3**^ **)**	155 ± 42	185 ± 35	160 ± 35	**<.001** ^**d**^ ^**,**^ ^**e**^	**<.001** ^**d**^ ^**,**^ ^**e**^	.07
**Tb.N (1/mm)**	1.25 ± 0.24	1.39 ± 0.20	1.21 ± 0.20	**<.001** ^**d**^ ^**,**^ ^**e**^	**<.001** ^**d**^ ^**,**^ ^**e**^	.07
**Tb.Th (mm)**	0.26 ± 0.02	0.26 ± 0.02	0.26 ± 0.02	.30	.27	.44
**Tb.Sp (mm)**	0.80 ± 0.17	0.69 ± 0.12	0.81 ± 0.15	**<.001** ^**d**^ ^**,**^ ^**e**^	**<.001** ^**d**^ ^**,**^ ^**e**^	**.045** ^**e**^
**Tb.1/N.SD (mm)**	0.33 ± 0.10	0.28 ± 0.08	0.34 ± 0.09	**<.001** ^**d**^ ^**,**^ ^**e**^	**<.001** ^**d**^ ^**,**^ ^**e**^	.12
**Ct.Ar (mm** ^**2**^ **)**	161 ± 32	147 ± 26	141 ± 26	**<.001** ^**c**^ ^**,**^ ^**e**^	**<.001** ^**c**^ ^**,**^ ^**e**^	**<.001** ^**c**^ ^**,**^ ^**e**^
**Ct.vBMD (mg HA/cm** ^**3**^ **)**	829 ± 91	796 ± 70	844 ± 76	**<.001** ^**d**^ ^**,**^ ^**e**^	**.002** ^**d**^ ^**,**^ ^**e**^	.09
**Ct.Po (%)**	4.39 ± 1.96	4.46 ± 1.53	3.76 ± 1.59	**.01** ^**d**^	**.03** **^f^**	.22
**Ct.Th (mm)**	1.64 ± 0.32	1.53 ± 0.28	1.48 ± 0.29	**.004** ^**c**^	**0.001** ^**c**^ ^**,**^ ^**e**^	**.001** ^**c**^ ^**,**^ ^**e**^
**Tb. Plate number**	1.51 ± 0.14	1.60 ± 0.14	1.53 ± 0.12	**<.001** ^**d**^ ^**,**^ ^**e**^	**<.001** ^**d**^ ^**,**^ ^**e**^	.05
**Tb. Rod number**	1.44 ± 0.18	1.48 ± 0.19	1.40 ± 0.15	**0.01** ^**d**^	**.01** ^**d**^	.29
**Plate-rod junction density**	3.44 ± 1.05	3.94 ± 1.16	3.29 ± 0.90	**<.001** ^**d**^ ^**,**^ ^**e**^	**<.001** ^**d**^ ^**,**^ ^**e**^	.21
**Plate-plate junction density**	2.61 ± 0.74	3.09 ± 0.80	2.61 ± 0.64	**<.001** ^**d**^ ^**,**^ ^**e**^	**<.001** ^**d**^ ^**,**^ ^**e**^	.09
**Stiffness**	221 424 ± 51 634	221 538 ± 43 109	206 272 ± 42 002	**.03** ^**f**^	**.01** ^**c**^ ^**,**^ ^**d**^	.15
**Failure load (N)**	11 938 ± 2676	11 892 ± 2238	11 095 ± 2173	**.03** ^**d**^	**.01** ^**c**^ ^**,**^ ^**d**^	.11

a Model adjusted for age, BMI, calcium intake, smoking, diabetes, liver disease, and HIV.

b Model adjusted for age, BMI, calcium intake, smoking, diabetes, liver disease, HIV, education, and household income.

c *p* < 0.05 CH vs NHB.

d *p* < 0.05 CH vs NHW.

e *p* < 0.05 NHB vs NHW.

f No pairwise Tukey comparisons had *p* < 0.05.

Data are shown as mean ± SD. Values of *p* < .05 were considered significant and are shown in bold.

Abbreviations: HA, hydroxyapatite; CH, Caribbean Hispanic; NHB, non-Hispanic black; NHW, non-Hispanic white.

Compared to NHB, CH men had 8.2% lower total area (*p* = .003) with a 14.1% smaller (*p* < .001) and 10.8% thinner (*p* = .004) cortex before adjustment. After adjustment, CH men still had 8.6% lower total area (model 1; *p* = .01). CH men also had 16.2% smaller cortical area (*p* < .001) and a 12.0% thinner cortex (*p* = .001; model 1). Adjusted stiffness and failure load were 10.8%-11.0% lower (*p* = .01) for CH men (model 1).

Compared to NHW men, NHB men had worse trabecular vBMD and microstructure prior to adjustment of covariates. NHB men had 11.3%-19.7% lower trabecular number and density with 13.5%-16.0% more widely spaced and heterogeneously distributed trabeculae (all *p* < .001). Before adjustment, NHB men had 4.0%-8.5% higher cortical density and area (*p* < .001). By ITS, NHB men had 5.9% fewer trabecular plates and 14.6%-18.5% lower plate-rod and plate-plate junctions (all *p* < .001) before adjustment. After adjustment for age, BMI, calcium intake, smoking, diabetes, liver disease, and HIV (model 1), all differences persisted. Cortical thickness was also 7.3% higher (Figure 2B) in NHB vs NHW men. There was no difference in stiffness and failure load before or after adjustment of covariates.

Including the current use of osteoporosis medication as a covariate in model 1 did not change the significance of results. Replacing BMI with weight and including height as covariate, differences in total area, cortical porosity, stiffness, and failure load were attenuated. After correcting for multiple comparisons for the 4 trabecular parameters noted above, all differences remained significant (*p* < .0125).

#### Diaphyseal tibia

Table 5 and Figure 2C show differences in skeletal microstructure at the diaphyseal tibia by HR-pQCT. Compared to NHW, CH men had 10.7%-11.9% smaller and thinner cortices, with 2.5% higher density and 11.1%-11.5% lower failure load and stiffness (all *p* < .001) prior to adjustment. After adjustment for age, BMI, calcium intake, smoking, diabetes, liver disease, and HIV, differences remained significant (Figure 2C).

**Table 5:** Racial/ethnic differences in vBMD, microstructure, and estimated mechanical competence by HR-pQCT at the diaphyseal tibia scanned at 30% relative offset.

**Parameter**	**NHB (*n* = 63)**	**NHW (*n* = 90)**	**CH (*n* = 102)**	***p***	**Adjusted *p*** ^**a**^	**Adjusted *p*** ^**b**^
**Ct.Ar (mm** ^**2**^ **)**	309 ± 62	299 ± 37	270 ± 42	**<.001** ^**c**^ ^**,**^ ^**d**^	**<.001** ^**c**^ ^**,**^ ^**d**^	**<.001** ^**c**^ ^**,**^ ^**d**^
**Ct.vBMD (mg HA/cm** ^**3**^ **)**	1011 ± 46	999 ± 35	1025 ± 39	**<.001** ^**c**^	**<.001** ^**c**^	**.02** ^**c**^
**Ct.Po (%)**	1.10 ± 1.13	1.39 ± 0.79	1.10 ± 0.74	**.04** ^**f**^	.05	.09
**Ct.Th (mm)**	5.70 ± 1.08	5.87 ± 0.69	5.24 ± 0.81	**<.001** ^**c**^ ^**,**^ ^**d**^	**<.001** ^**c**^ ^**,**^ ^**d**^	**.004** ^**c**^ ^**,**^ ^**d**^
**Stiffness**	332 781 ± 61 483	318 791 ± 40 714	285 819 ± 44 540	**<.001** ^**c**^ ^**,**^ ^**d**^	**<.001** ^**c**^ ^**,**^ ^**d**^	**<.001** ^**c**^ ^**,**^ ^**d**^
**Failure load (N)**	18 798 ± 3583	17 870 ± 2280	16 084 ± 2548	**<.001** ^**c**^ ^**,**^ ^**d**^	**<.001** ^**c**^ ^**,**^ ^**d**^	**<.001** ^**c**^ ^**,**^ ^**d**^

a Model adjusted for age, BMI, calcium intake, smoking, diabetes, liver disease, and HIV.

b Model adjusted for age, BMI, calcium intake, smoking, diabetes, liver disease, HIV, education, and household income.

c *p* < 0.05 CH vs NHW.

d *p* < 0.05 CH vs NHB.

e *p* < 0.05 NHB vs NHW.

f No pairwise Tukey comparisons had *p* < 0.05.

Data are shown as mean ± SD. Values of *p* < 0.05 were considered significant and are shown in bold.

Abbreviations: HA, hydroxyapatite; CH, Caribbean Hispanic; NHB, non-Hispanic black; NHW, non-Hispanic white.

Compared to NHB men, CH men had 8.8%-14.6% thinner and smaller cortices, with 16.4%-16.9% lower stiffness and failure load (all *p* < .001) prior to adjustment. Differences persisted after adjustment (model 1), with 10.2%-15.2% adjusted thinner/smaller cortices and 16.6%-17.0% lower adjusted stiffness and failure load (all *p* < .001). There were no differences between NHW and NHB before or after adjustment for covariates (model 1; Figure 2C).

**Figure 3:**
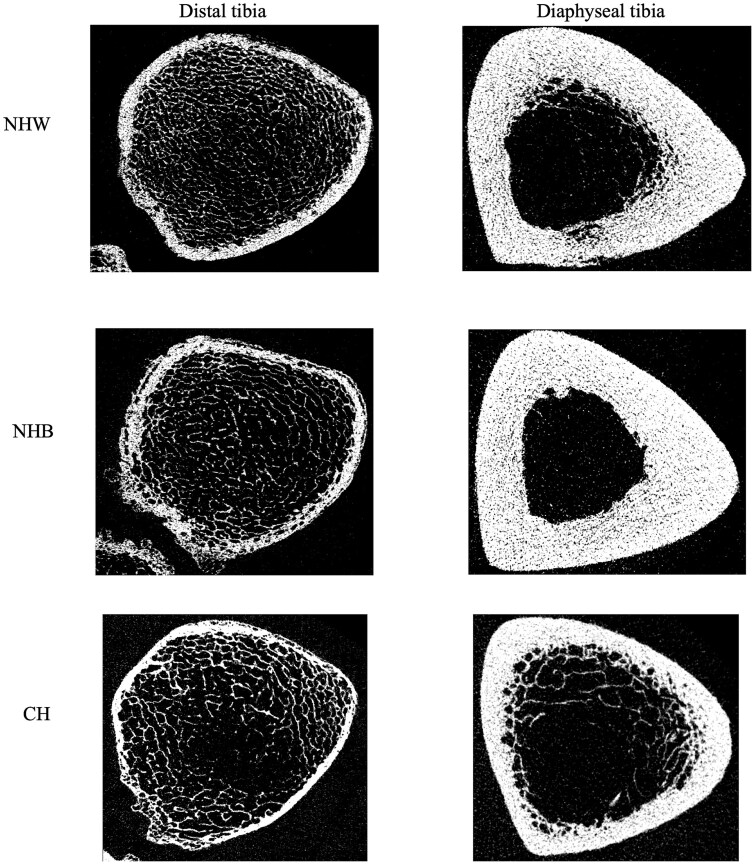
Representative HR-pQCT images of the distal tibia (left) and diaphyseal tibia (right) in NHW (top), NHB (middle), and CH (bottom).

Results did not change after including current osteoporosis treatment as a covariate in adjusted models. Adjusting for height and weight instead of BMI (Table S1), cortical porosity was now significantly lower (*p* = .04). Representative HR-pQCT images at both the distal and diaphyseal tibia for NHW, NHB, and CH men are shown in Figure 3.

### Adjustment for income and education

As shown in Table 2, there were no differences in aBMD between NHW and CH men after adjusting for socioeconomic (SE) factors (model 2). Compared to NHB, CH men had lower aBMD at the FN (*p* < .001) and 1/3 radius (*p* < .001) after adjustment. Differences in aBMD between NHB and NHW men persisted adjusting for income and education (model 2). NHB had higher aBMD at the FN (*p* < .001), 1/3 radius (*p* < .001), and TH (*p* = .05) compared to NHW men.

All differences by HR-pQCT at the radius were no longer significant after adjusting for education and income (Table 3). At the distal tibia, differences between NHW and CH men were attenuated and no longer significant. Cortical advantages (Ct.Th and Ct.Ar) in NHB men compared to NHW and CH men persisted but differences in mechanical competence were no longer significant. CH men still had lower total area (*p* = .02) compared to NHB men. Differences in trabecular spacing remained significant in NHB compared to NHW men (Table 4; *p* = .045). However, after correcting for multiple comparisons for the four correlated trabecular parameters noted above, trabecular spacing was not significant (*p* > .0125). At the diaphyseal tibia, all differences, including stiffness and failure load, remained significant for CH men compared to NHW and NHB men after adjustment for SE factors (Table 5). After including osteoporosis treatment as an additional covariate in model 2, all results were unchanged. After including height and weight instead of BMI, differences in total area at the distal tibia and cortical area at the diaphyseal tibia were attenuated and no longer significant.

## Discussion

We believe this is the first study to assess racial differences in skeletal microstructure by HR-pQCT in elderly men. We found that elderly CH men from a population-based study had lower mechanical competence at the tibia compared to both NHW and NHB men. The particular microstructural differences associated with lower stiffness and failure load, however, varied by tibial location and racial/ethnic comparison. CH men had consistently worse trabecular microstructure across most indices at the distal tibia compared to NHW men, leading to lower mechanical competence. CH men also had lower stiffness and failure load compared to NHB men, but this was due to smaller bone size and lower cortical thickness. Disadvantageous cortical features were also seen at the diaphyseal tibia in CH men compared to both NHW and NHB men, including lower Ct.Ar and Ct.Th leading to lower mechanical competence. These results are important because lower tibial mechanical competence as measured by μFEA of HR-pQCT images has been shown in other studies (that include predominantly NHW women) to predict incident fracture.^13^ Specifically, failure load and stiffness were more strongly associated with incident fracture than areal BMD.^13^ Thus, based on the μFEA results, we would anticipate that fracture risk is higher in CH compared to NHW or NHB men. However, it is important to confirm that μFEA indices are similarly predictive in under-represented minorities in future studies.

There were some site-specific differences. At the radius, some trabecular indices were worse at the radius in elderly CH compared to NHW men, but ultimately mechanical competence did not differ compared to either NHW or NHB men. The reasons for site-specific differences are not clear, but studies utilizing HR-pQCT have often shown greater differences at the tibia. We did not find differences in stiffness or failure load between NHW and NHB at any site as the worse trabecular indices in NHB men were offset by better cortical indices.

Some of the results in elderly men are similar to those found in elderly women from the same cohort. For example, CH women had lower stiffness and failure load compared to NHB women, though differences were more pervasive at both radius and tibia in women.^14^ In contrast, mechanical competence did not differ between CH and NHW at any site while NHB women had higher mechanical competence at all sites compared to CH women. Our data suggest racial differences in mechanical competence may be sex-specific.

There are no comparable racially diverse HR-pQCT data in elderly males. In the Osteoporotic Fractures in Men study (Mr. OS), which used central QCT to assess racial differences in hip vBMD, Hispanic men did not differ from NHW men. Compared to HR-pQCT studies in male adolescents and younger men, our results differ in some ways. Bone strength in studies using pQCT and HR-pQCT has been demonstrated to be higher in NHB than NHW children and younger adults.^31^^,^^32^ We did not find this to be true in older men. It is possible that vBMD and microstructure differences become less marked in older age. However, QCT data from the MrOS study also showed that older NHB men had greater hip trabecular and cortical vBMD compared to NHW men.^33^ Similar to the current study, a study of CH young men from our institution also showed similar mechanical competence compared to NHW young men.^34^

We explored the effect of adjusting for SE factors upon HR-pQCT and μFEA indices. Many of the differences at the radius and distal tibia were attenuated and no longer significant after adjusting for such factors, particularly those between CH and NHW participants. This finding may suggest that some racial differences in bone quality are modifiable, but this requires further study. The results could suggest that poorer skeletal microstructure may be preventable with more education and better income, though these may also be a proxy for other factors, such as a healthier diet. We also explored adjusting for height and weight in place of BMI. Results were similar, though some differences including geometry, stiffness and failure load were attenuated at the tibia.

Our results with regard to aBMD by DXA are similar to most other studies showing NHB men have higher aBMD compared to NHW^35–38^ and Hispanic men (of different Hispanic origins).^35^ We found no differences in aBMD between CH and NHW men. Prior studies have shown variable results, depending on the population and their origin. A population-based study in older men (Mr. OS) showed Hispanic (origin not reported) men (only 2.1% of study population) had higher hip but lower spine aBMD compared to NHW men.^37^ An interesting study by Araujo et al. showed higher aBMD in Hispanics (of different origins but including CH groups) was only present in younger but not older age, suggesting differences may dissipate with aging.^35^ Other studies have shown lower aBMD in Hispanic (Mexican) vs NHW men.^3^^,^^39–41^

This study has some limitations. Even though this analysis is the only study assessing racial differences in skeletal microstructure and mechanical competence using HR-pQCT and μFEA in elderly men and is comparable in size to HR-pQCT studies in women, it is still relatively small when compared to population-based aBMD studies. Power to detect differences, particularly between the NHW and NHB men was limited. With the current sample size, we had 80% power with a 2-tailed alpha of 5% to detect a 0.46SD difference in FL at the radius and tibia between NHW and NHB men. We did not find differences in mechanical indices at the radius between any groups. With the current sample size, we had 80% power with a 2-tailed alpha of 5% to detect a 0.45SD difference in FL at the radius between CH and NHB men and about a 0.41SD difference in FL at the radius between CH and NHW men. We could have failed to detect smaller differences. In this cross-sectional study, we were not able to assess racial/ethnic differences in incident fracture. Multiple testing can cause Type I errors, but the results remained consistent after adjustment for multiple comparisons among several highly correlated HRpQCT parameters. Although patient interviews were rigorous, there is potential for recall bias. Despite these limitations, we believe the results are important and impactful for several reasons. We were able to detect differences in mechanical competence between CH men and the other 2 groups at the tibia. This is the first study assessing racial differences in non-Hispanic White, non-Hispanic Black, and Caribbean Hispanic elderly men using HR-pQCT. Ultimately, our study addresses a gap in research, which benefits individuals who are historically underrepresented in skeletal research. We are not able to evaluate incident fracture risk.

This study has numerous strengths. Since we enrolled participants from a population-based cohort study, we were able to limit potential selection bias and increase the generalizability of our results. We used several tools to evaluate musculoskeletal health and obtained extensive information on skeletal covariates. Scanning participants at the diaphyseal tibial site allowed us to obtain increased understanding of racial differences in cortical bone. We used a second-generation HR-pQCT instrument, which has better resolution than the first-generation machine. A relative offset to assess parameters ensured that the same region of interest was evaluated across groups with varying mean height.

In conclusion, elderly CH men have lower skeletal mechanical competence at the tibia compared to NHW and NHB men. These characteristics may confer an increased risk of incident fracture for CH men. Some of the differences between CH and NHW may be related to modifiable SE factors. Studies assessing the ability of HR-pQCT indices to predict incident fracture, as well as how SE factors affect fracture risk, are needed in individuals historically underrepresented in skeletal research.

## Supplementary Material

Supplemental_Table_ziaf110

## Data Availability

The data sets generated during and/or analyzed during the current study are not publicly available but are available from the corresponding author on reasonable request.
