# Supplementary material for: Elderly Caribbean Hispanic men have lower tibial stiffness and failure load compared to non-Hispanic White and Black men
Source: JBMR Plus. 2025 Jun 24;9(9):ziaf110. doi: 10.1093/jbmrpl/ziaf110 (PMC12342823; doi:10.1093/jbmrpl/ziaf110)
Supplement: Supplemental_Table_ziaf110 [file supplemental_table_ziaf110.docx]

| **Supplemental Table 1. Racial/Ethnic Differences in aBMD, and TBS by DXA, vBMD, Microstructure, and Estimated Mechanical Competence by HR-pQCT.** | | | | | | |
| --- | --- | --- | --- | --- | --- | --- |
| **Parameter** | **NHB (*n* = 63)** | **NHW (*n* = 90)** | **CH (*n* = 102)** | ***p*** | **Adjusted *p*^#^** | **Adjusted *p*^##^** |
| **DXA** | | | | | | |
| LS *T*-score | 0.4 ± 2.0 | 0.4 ± 1.8 | 0.1 ± 1.7 | 0.59 | 0.91 | 0.84 |
| FN *T*-score | -0.7 ± 1.1 | -1.3 ± 0.9 | -1.1 ± 1.1 | **0.002^c^** | **0.01^c^** | **0.02^c^** |
| TH *T*-score | -0.5 ± 1.0 | -0.8 ± 0.9 | -0.5 ± 1.0 | **0.04*** | 0.07 | 0.11 |
| 1/3-Radius *T*-score | -0.3 ± 1.6 | -1.3 ± 1.4 | -1.0 ± 1.4 | **<0.001^b,c^** | **0.006^c^** | **0.01^c^** |
| TBS | 1.3 ± 0.1 | 1.3 ± 0.1 | 1.3 ± 0.1 | 0.21 | 0.49 | 0.74 |
| **Distal Radius (4%) by HRpQCT** | | | | | | |
| Tt.Ar (mm^2^) | 387 ± 59 | 366 ± 63 | 360 ± 49 | **0.01^b^** | 0.27 | 0.88 |
| Tot.vBMD (mg HA/cm^3^) | 277 ± 61 | 282 ± 59 | 289 ± 64 | 0.42 | 0.77 | 0.37 |
| Tb.vBMD (mg HA/cm^3^) | 153 ± 35 | 167 ± 37 | 159 ± 37 | 0.07 | 0.16 | 0.79 |
| Tb.N (1/mm) | 1.38 ± 0.20 | 1.48 ± 0.20 | 1.34 ± 0.21 | **<0.001^a,c^** | **<0.001^a^** | 0.22 |
| Tb.Th (mm) | 0.24 ± 0.02 | 0.24 ± 0.02 | 0.24 ± 0.02 | **0.04^a^** | 0.07 | 0.13 |
| Tb.Sp (mm) | 0.70 ± 0.12 | 0.65 ± 0.11 | 0.72 ± 0.14 | **<0.001^a,c^** | **<0.001^a^** | 0.23 |
| Tb.1/N.SD (mm) | 0.27 ± 0.07 | 0.25 ± 0.07 | 0.29 ± 0.11 | **0.01^a^** | **0.002^a,b^** | 0.07 |
| Ct.Ar (mm^2^) | 72 ± 16 | 65 ± 14 | 69 ± 15 | **0.03^c^** | 0.05 | 0.07 |
| Ct.vBMD (mg HA/cm^3^) | 826 ± 75 | 811 ± 69 | 837 ± 73 | **0.04^a^** | 0.13 | 0.35 |
| Ct.Po (%) | 1.46 ± 0.89 | 1.50 ± 0.75 | 1.37 ± 0.79 | 0.52 | 0.58 | 0.86 |
| Ct.Th (mm) | 1.04 ± 0.24 | 0.97 ± 0.21 | 1.04 ± 0.25 | 0.07 | 0.21 | 0.09 |
| Tb. Plate number | 1.52 ± 0.17 | 1.55 ± 0.17 | 1.53 ± 0.16 | 0.48 | 0.32 | 0.85 |
| Tb. Rod number | 1.59 ± 0.18 | 1.62 ± 0.18 | 1.55 ± 0.17 | **0.03^a^** | **0.01^a^** | 0.28 |
| Plate-rod junction density | 4.16 ± 1.23 | 4.44 ± 1.27 | 3.97 ± 1.07 | **0.03^a^** | **0.01^a^** | 0.48 |
| Plate-plate junction density | 2.75 ± 0.86 | 2.94 ± 0.93 | 2.73 ± 0.79 | 0.20 | 0.09 | 0.85 |
| Stiffness | 77412 ± 20674 | 73001 ± 20674 | 75817 ± 21288 | 0.42 | 0.21 | 0.16 |
| Failure load (N) | 4190 ± 1080 | 3942 ± 1098 | 4072 ± 1138 | 0.40 | 0.23 | 0.17 |
| **Distal Tibia (7.3%) by HRpQCT** | | | | | | |
| Tt.Ar (mm^2^) | 892 ± 135 | 862 ± 136 | 824 ± 105 | **0.003^b^** | 0.80 | 0.85 |
| Tot.vBMD (mg HA/cm^3^) | 275 ± 56 | 289 ± 48 | 277 ± 49 | 0.14 | 0.06 | 0.86 |
| Tb.vBMD (mg HA/cm^3^) | 155 ± 42 | 185 ± 35 | 160 ± 35 | **<0.001^a,c^** | **<0.001^a,c^** | 0.10 |
| Tb.N (1/mm) | 1.25 ± 0.24 | 1.39 ± 0.20 | 1.21 ± 0.20 | **<0.001^a,c^** | **<0.001^a,c^** | 0.07 |
| Tb.Th (mm) | 0.26 ± 0.02 | 0.26 ± 0.02 | 0.26 ± 0.02 | 0.30 | 0.38 | 0.57 |
| Tb.Sp (mm) | 0.80 ± 0.17 | 0.69 ± 0.12 | 0.81 ± 0.15 | **<0.001^a,c^** | **<0.001^a,c^** | **0.044**^*^ |
| Tb.1/N.SD (mm) | 0.33 ± 0.10 | 0.28 ± 0.08 | 0.34 ± 0.09 | **<0.001^a,c^** | **<0.001^a,c^** | 0.12 |
| Ct.Ar (mm^2^) | 161 ± 32 | 147 ± 26 | 141 ± 26 | **<0.001^b,c^** | **0.01^b,c^** | **0.006^b,c^** |
| Ct.vBMD (mg HA/cm^3^) | 829 ± 91 | 796 ± 70 | 844 ± 76 | **<0.001^a,c^** | **0.002^a,c^** | 0.11 |
| Ct.Po (%) | 4.39 ± 1.96 | 4.46 ± 1.53 | 3.76 ± 1.59 | **0.01^a^** | 0.12 | 0.53 |
| Ct.Th (mm) | 1.64 ± 0.32 | 1.53 ± 0.28 | 1.48 ± 0.29 | **0.004^b^** | **0.01^b^** | **0.008^b,c^** |
| Tb. Plate number | 1.51 ± 0.14 | 1.60 ± 0.14 | 1.53 ± 0.12 | **<0.001^a,c^** | **<0.001^a,c^** | 0.14 |
| Tb. Rod number | 1.44 ± 0.18 | 1.48 ± 0.19 | 1.40 ± 0.15 | **0.01^a^** | **0.01^a^** | 0.26 |
| Plate-rod junction density | 3.44 ± 1.05 | 3.94 ± 1.16 | 3.29 ± 0.90 | **<0.001^a,c^** | **<0.001^a,c^** | 0.21 |
| Plate-plate junction density | 2.61 ± 0.74 | 3.09 ± 0.80 | 2.61 ± 0.64 | **<0.001^a,c^** | **<0.001^a,c^** | 0.15 |
| Stiffness | 221424 ± 51634 | 221538 ± 43109 | 206272 ± 42002 | **0.03*** | 0.42 | 0.85 |
| Failure load (N) | 11938 ± 2676 | 11892 ± 2238 | 11095 ± 2173 | **0.03^a^** | 0.44 | 0.82 |
| **Diaphyseal Tibia (30%) by HRpQCT** | | | | | | |
| Ct.Ar (mm^2^) | 309 ± 62 | 299 ± 37 | 270 ± 42 | **<0.001^a,b^** | **0.03^a^** | 0.06 |
| Ct.vBMD (mg HA/cm^3^) | 1011 ± 46 | 999 ± 35 | 1025 ± 39 | **<0.001^a^** | **<0.001^a^** | **0.03^a^** |
| Ct.Po (%) | 1.10 ± 1.13 | 1.39 ± 0.79 | 1.10 ± 0.74 | **0.04*** | **0.04*** | 0.10 |
| Ct.Th (mm) | 5.70 ± 1.08 | 5.87 ± 0.69 | 5.24 ± 0.81 | **<0.001^a,b^** | **<0.001^a,b^** | **0.03^a^** |
| Stiffness | 332781 ± 61483 | 318791 ± 40714 | 285819 ± 44540 | **<0.001^a,b^** | **0.006^a,b^** | **0.03^b^** |
| Failure load (N) | 18798 ± 3583 | 17870 ± 2280 | 16084 ± 2548 | **<0.001^a,b^** | **0.01^a,b^** | **0.03^b^** |
| *Note*: Data are shown as mean ± SD or %. Values of *p* < 0.05 were considered significant and are shown in bold.  Abbreviations: CH = Caribbean Hispanic; NHB = non-Hispanic Black; NHW = non-Hispanic White.  ^a^*p* < 0.05 CH versus NHW.  ^b^*p* < 0.05 CH versus NHB.  ^c^*p* < 0.05 NHB versus NHW.  ^#^Model adjusted for age, height, weight, calcium intake, smoking, diabetes, liver disease, HIV.  ^##^Model adjusted for age, height, weight, calcium intake, smoking, diabetes, liver disease, HIV, education, household income.  *No pairwise Tukey comparisons had p<0.05. | | | | | | |
